# Identification of MXRA5 as a novel biomarker in colorectal cancer

**DOI:** 10.3892/ol.2012.1038

**Published:** 2012-11-21

**Authors:** GUANG-HUI WANG, LING YAO, HONG-WEI XU, WEN-TAO TANG, JI-HONG FU, XIAO-FANG HU, LONG CUI, XUE-MIN XU

**Affiliations:** 1 Colorectal Surgery Department, Xinhua Hospital, School of Medicine, Shanghai Jiao Tong University, Shanghai 200092;; 2 School of Life Sciences and Biotechnology, Shanghai Jiao Tong University, Shanghai 200092;; 3 School of Biomedical Engineering and Med-X Research Institute, Shanghai Jiao Tong University, Shanghai 200092;; 4 Department of Gastroenterology, Kunshan Hospital of Traditional Chinese Medicine, Jiangsu 215300, P.R. China

**Keywords:** MXRA5, colorectal cancer, proteomics, omental metastasis

## Abstract

In our previous study, significantly high expression levels of matrix-remodeling associated 5 (MXRA5) were identified in fresh-cultured colorectal cancer (CRC) tissues compared with their normal adjacent mucosa by differential secretome analysis. Whether MXRA5 is a potential serum biomarker of CRC has not been evaluated. The aim of this study was to investigate the association between MXRA5 expression and clinicopathological characteristics of CRC patients. The MXRA5 expression levels were determined by quantitative real-time PCR (qRT-PCR) and immunohistochemistry (IHC) in 20 colorectal adenoma tissues, 156 CRC tissues and their corresponding adjacent normal mucosa. Relative quantity (RQ) value and immunoreactive score (IRS) were used for quantitative assessment. The staining for MXRA5 protein was mainly located in the cytoplasm of CRC cells. All CRC tissues were positively stained, with a higher expression rate (IRS>4) of 67% (105/156), and a lower expression rate (IRS≤4) of 33% (51/156). Meanwhile, their corresponding normal tissues exhibited little positive staining; the higher expression rate was 0% (0/156) and the lower expression rate was 25% (16/156). Additionally, more than half of the adenoma tissues were positively stained; the higher expression rate was 15% (3/20) and the lower expression rate was 50% (10/20). The MXRA5 protein positive staining rates were significantly correlated with the lesion sites (colon vs. rectum, 76 vs. 59%), TNM staging (I+II vs. III+IV, 56 vs. 73%) and metastasis (present vs. absent; 76 vs. 61%) with the most high positive staining rate observable in omental metastasis (82%). However, MXRA5 mRNA expression levels showed no significant differences between CRC tissues and their corresponding normal tissues, and no significant correlation between IRS and corresponding RQ value was observed. In this study, we present the first evaluation of MXRA5 protein expression in CRC tissue. Our results revealed that MXRA5 protein is aberrantly expressed in CRC tissues, and has potential value in early detection of CRC and prediction of omental metastasis.

## Introduction

Colorectal cancer (CRC) was the third most common type of malignancy and the third leading cause of cancer-related mortality (for both genders) worldwide in 2011 (1). At present, the pathogenesis of colorectal cancer remains unclear, and early detection remains the most promising approach to improving long-term survival of patients with CRC (2–4). Numerous molecular markers, including the carcinoembryonic antigen (CEA), have been used for detecting CRC (5,6). However, these biomarkers do not provide sufficient sensitivity and reliability for the detection of CRC (7,8). Thus, there is an urgent demand for the detection of new biomarkers that are capable of serving as diagnostic and prognostic markers for CRC.

With the invention and development of mass spectrometry (MS), proteomics analysis is currently considered to be a strong tool for global evaluation of protein expression and has been widely applied in analysis of diseases, particularly in cancer research (9–12). In our previous study, we compared the secretome of fresh-cultured colorectal tissues and paired normal colorectal tissues. By adopting the proteomics strategy of one-dimensional gel electrophoresis coupled with liquid chromatography-tandem mass spectrometry and quantification with label-free spectral counting, 123 differentially expressed secreted proteins (DESPs) were identified. One of the top 10 upregulated DESPs, EFEMP2, was validated as a serum biomarker in the early stage of CRC (13).

In the present study, another protein, matrix-remodeling associated (MXRA) protein, was selected for investigation into tumorigenesis in CRC, as few previous studies have included this protein.

## Materials and methods

### Tissue samples

In our study, 176 tissue samples were used, including 156 CRC tissues and paired normal tissues from CRC patients who had not received pre-operative chemotherapy or radiotherapy, and 20 adenoma tissues from the control subjects. For the immunohistochemistry (IHC) experiment, one set of these tissues was cut into formalin-fixed, paraffin-embedded tissue blocks. For the quantitative real-time PCR (qRT-PCR) experiment, the other set of these tissues was immediately frozen in liquid nitrogen and then stored at −80°C until use. Tumor staging was classified according to the TNM classification system (UICC). The clinical features of these tissue samples are described in Table I. All tissue samples were obtained between September 2009 and October 2010 in the Xinhua Hospital Affiliated to Shanghai Jiaotong University School of Medicine. The present study was approved by the Xinhua Hospital Ethics Committee and conducted with the consent of all patients.

### IHC and tissue microarray

All tissues mentioned previously were made into sections and two tissue microarrays. The sections were stained with hematoxylin and eosin (H&E) to ensure that the sectioned block contained either normal or tumor cells. Other sections were then stained immunohisto-chemically. First, sections were deparaffinized in xylene and then rehydrated in a series of ethanol solutions of increasing strength. In order to increase specificity and sensitivity, sections were pretreated by microwaving for 5 min on high mode and then 10 min on middle mode in citrate buffer, pH 6.5. Peroxidase activity was blocked with 3% H_2_O_2_-methanol for 30 min and sections were incubated with normal goat serum for 30 min to eliminate non-specific staining. Sections were incubated with anti-human MXRA5 polyclonal antibodies diluted at 1:10 (SAB1402656, Sigma, St. Louis, MO, USA) overnight at 4°C. Then, sections were washed 3 times with phosphate-buffered saline (PBS) and incubated with secondary antibody (GK500705; Gene Company Ltd., Shanghai, China) for another 30 min at room temperature. Following three 5-min rinses in PBS, staining was completed with 10 min incubation with 3,3′-diaminobenzidine (DAB) solution. Finally, sections were counterstained with 0.1% hematoxylin and coverslipped.

For the assessment, five representative fields were assessed per section at ×200 magnification with a light microscope (Carl Zeiss, Göttingen, Germany). The immunostaining was evaluated according to the following standards; staining intensity was classified as 0 (lack of staining), 1 (mild staining), 2 (moderate staining) or 3 (strong staining), and the percentage of staining was designated 1 (<25%), 2 (25–50%), 3 (51–75%) or 4 (>75%). For each section, the semi-quantitative score was calculated by multiplying these two values (which ranged from 0–12) and the result was defined as either negative (0), weakly positive (1–3), positive (4–7) or strongly positive (8–12). Two histopathologists blindly reviewed the slides and evaluated the data.

### RNA extraction and qRT-PCR

Among the 156 CRC tissues, 70 CRC tissues and their matched normal tissues were randomly selected for RQ value detection of MXRA5 mRNA expression. Total RNA was extracted from frozen tissues with the RNAiso Plus kit (Takara Bio Inc., Shiga, Japan) according to the manufacturer’s instructions. Reverse transcription of extracted RNA (500 ng) was performed using RNase H-deficient reverse transcriptase (Superscript II; Life Technologies, Carlsbad, CA, USA). The reverse transcription reaction mixture (2 μl) was used for quantification of MXRA5 gene expression by RT-PCR assay. Gene specific primers used in qRT-PCR were as follows: MXRA5 forward primer, 5′-CAT TGC TAG ACA CGT GGA AAG A-3′; reverse primer, 5′-TCT CAT TGC CGT GAA TCA TAA G-3′. qRT-PCR was conducted in an Applied Biosystems 7500 Real-Time PCR system (Applied Biosystems, Foster City, CA, USA) using SYBR Premix Ex Taq™ kit (Takara) according to the manufacturer’s instructions. The reaction was repeated three times and threshold cycle numbers were averaged. The expression intensity of MXRA5 in CRC samples was expressed as fold changes over the average of normal tissue samples. GAPDH was amplified from the same RNA samples and served as an internal control.

### Statistical analysis

Statistical calculations were performed using SPSS 17.0 software (SPSS Inc., Chicago, IL, USA). Comparisons of data between two groups were analyzed using a Student’s t-test and a two-tailed P<0.05 was considered to indicate a statistically significant difference.

## Results

### IHC staining

**Figure 1.**
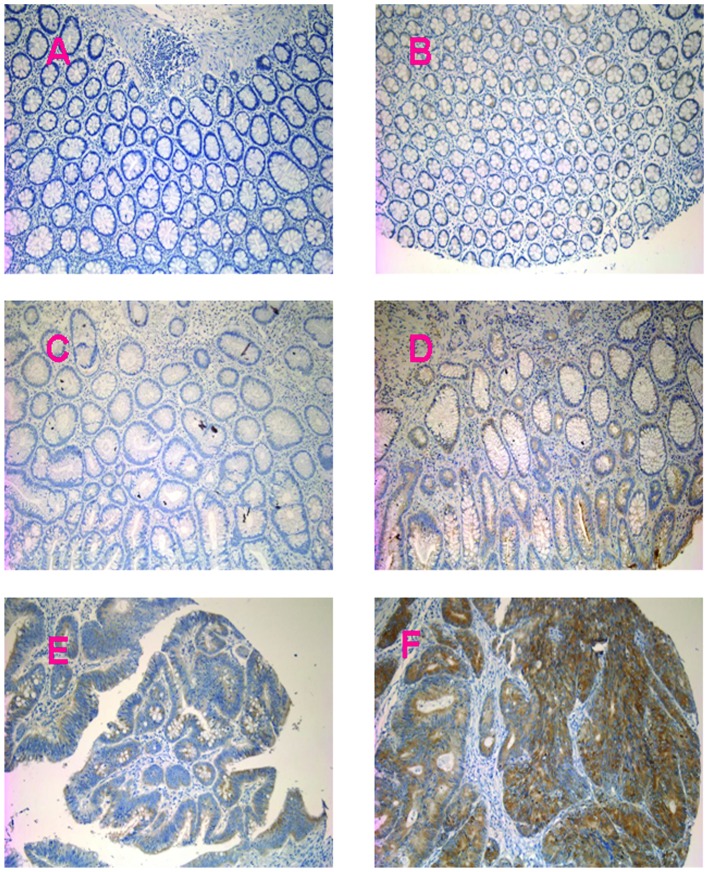
Representative immunohistochemical (IHC) staining for matrix remodeling associated 5 (MXRA5) expression. (A) Negative staining (score 0) in normal tissues. (B) Weakly positive staining (score 2) in normal tissues. (C) Negative staining (score 0) in adenoma tissues. (D) Weakly positive staining (score 2) in adenoma tissues. (E) Weakly positive staining (score 2) in colorectal cancer (CRC) tissues. (F) Strongly positive staining (score 12) in CRC tissues. Magnification, ×100.

To investigate the oncogenic properties of MXRA5 in CRC, paraffin-embedded tissues were stained aganinst MXRA5 antibody. As shown in Fig. 1, staining for MXRA5 was mainly located in the cytoplasm of CRC cells, suggesting that the CRC cells were responsible for the overexpression of MXRA5.

**Figure 2.**
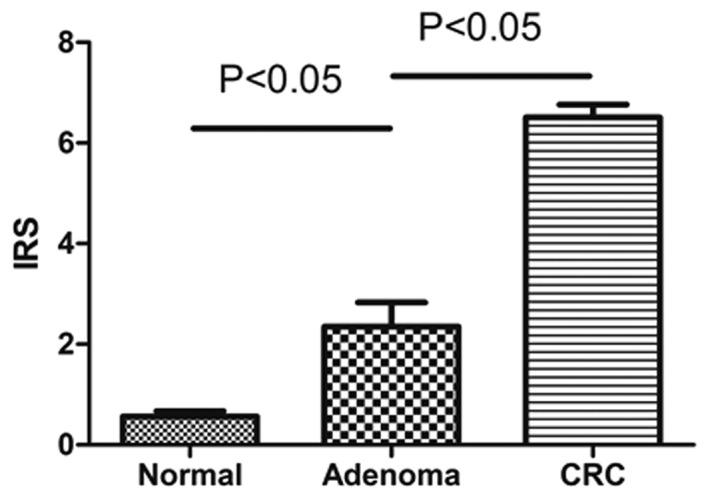
The immunoreactive score (IRS) of matrix remodeling associated 5 (MXRA5) protein expression in colorectal cancer (CRC) tissue, corresponding normal tissue and adenoma tissue.

As demonstrated in Table I, of 156 normal tissues, 84% (131/156) were negative (IRS, 0), while 16% (25/156) were weakly positive/positive (IRS, 1–4). Percentages of tissues exhibiting an IRS of 2, 3 and 4 were 0.6% (1/156), 6.4% (10/156) and 9% (14/156), respectively. In 20 adenoma specimens, scores of 0, 2, 3, 4 and 6 were detected in 35% (7/20), 20% (4/20), 15% (3/20), 15% (3/20) and 15% (3/20), respectively, while no strong staining (IRS, 9–12) was observed. Of the 156 CRC specimens, IRS of 2, 3, 4, 6, 8, 9 and 12 were detected in 8.3% (13/156), 10.9% (17/156), 13.5% (21/156), 30.8% (48/156), 16.0% (25/156), 3.8% (6/156) and 16.7% (26/156), respectively. There was a significant difference in MXRA5 expression in CRC tissues and adenoma tissues compared with normal tissues. Furthermore, as demonstrated in Fig. 2, the IRS of normal, adenoma and CRC tissue specimens were 0.56±1.31, 2.25±2.159 and 6.55±3.072, respectively. The IRS gradually increased from normal to adenoma to CRC tissue with significantly different expression between the different groups.

### Correlations with CRC characteristics

**Figure 3.**
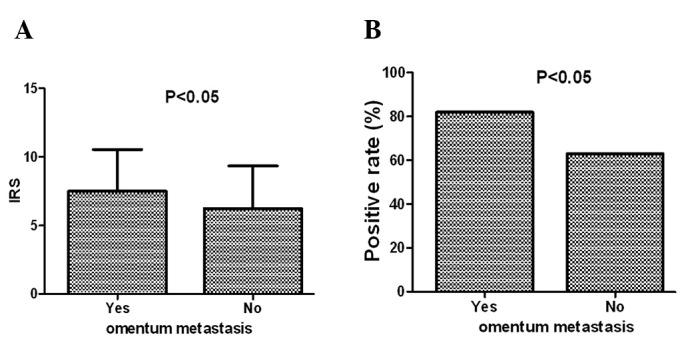
(A) The immunoreactive score (IRS) of matrix remodeling associated 5 (MXRA5) protein expression in patients with and without omental metastasis. (B) The rate of MXRA5 protein expression in patients with and without omental metastasis.

The correlation between MXRA5 staining and clinicopathological characteristics was investigated. As demonstrated in Table II, MXRA5 protein expression was significantly correlated with certain pathological features of CRC, including the lesion site of the right colon, advanced stage (III and IV) and distant metastasis, while no significant correlation was observed with gender, age, gross pathology, tumor diameter, differentiation, invasion or lymph metastasis. Furthermore, as sites of distant metastasis included the liver, pelvic cavity, omentum and other sites, the correlations of MXRA5 protein expression with the different types of distant metastasis were investigated in detail. As shown in Fig. 3, both the positive expression rate and IRS of MXRA5 expression were significantly higher in the omental metastasis group compared with the group without omental metastasis. No significant change was observed in the liver, pelvic cavity or any of the other sites of distant metastasis investigated.

### qRT-PCR analysis

Additionally, the differential expression of the MXRA5 gene in normal and CRC samples was detected by qRT-PCR analysis at the mRNA level. However, no significant differences in MXRA5 expression were found between tumor samples and patient-matched normal tissues. Furthermore, no significant correlation between the IRS of protein expression and its corresponding RQ value of mRNA expression was observed (data not shown).

## Discussion

MXRA5, also known as Adlican, is a 312-kDa protein and belongs to the MXRA gene family that participates in cell adhesion and matrix remodeling. MXRA2 is an α-parvin, a cell-matrix adhesion protein that co-localizes with actin filaments at membrane ruffles and focal contacts in fibroblasts (14). MXRA4 is a C1q complement component receptor. C1q has several functions, including stimulating endothelial expression of cell adhesion molecules and promoting cell attachment (15). MXRA5 is an adhesion proteoglycan with VEGF receptor activity that shows elevated expression in the cartilage of patients with osteoarthritis, and is involved in adhesion and matrix remodeling (16). Cell adhesion and matrix remodeling play a key role in many disease processes, including cancer, arthritis, angiogenesis, ulceration and fibrosis. Therefore, we hypothesized that MXRA genes may play an important role in tumor development.

Previous studies have revealed that the MXRA5 gene was upregulated in individuals exposed to fractionated radiation, by cDNA array (17,18). The gene has also been observed to be overexpressed in skin fibroblasts from centenarians compared with younger controls (19). Regarding its role in tumorigenesis, Buckanovich *et al*(20) found that MXRA5 was overexpressed in ovarian cancer compared with normal ovaries (by qRT-PCR) and it was involved in tumor angiogenesis. The authors simultaneously demonstrated that MXRA5 was absent in almost all normal colon tissues, with few exceptions. This is consistent with our results. The only study concerning MXRA5 in relation to CRC tumorigenesis was a study by Zou *et al*(21), which demonstrated using qRT-PCR that the MXRA5 gene was over-expressed in CRC tissues compared with their corresponding normal tissue, and that the gene may be involved in the development and progression of CRC (21). However, our data suggests that the protein expression of MXRA5 was aberrantly high in CRC tissues while the mRNA expression was not, which is inconsistent with the study by Zou *et al*. The reasons for this are mainly due to sample size; Zou *et al* only experimented with 13 CRC tissues, compared with our 156 CRC tissues for protein expression and 70 CRC tissues for mRNA expression.

Notably, MXRA5 protein expression was also detected in human adenoma tissues in our study, and the protein expression level of MXRA5 from normal colorectal to adenoma and then to carcinoma tissue markedly increased, paralleling the increasing severity of colorectal tissue injury. This result indicated that the aberrant protein expression of MXRA5 was an early event in CRC tumorigenesis.

Additionally, it was demonstrated that aberrant protein expression of MXRA5 was significantly correlated with the lesion site of CRC, advanced TNM stage and distant metastasis (omental metastasis in particular). The omentum is an important intraperitoneal structure with unique anatomic and pathologic features, mainly comprising of fat, while the numerous other components include blood vessels, lymphatics and cellular tissues of the immune system (22). Histologically, the omentum is composed of a double layer of peritoneum that extends inferiorly from the greater curvature of the stomach. Following extension for a distance that typically ranges from 14–36 cm, the greater omentum turns superiorly on itself to drape over the transverse colon and extend to the retroperitoneal pancreas. Metastatic disease involving the omentum is far more common than primary tumors. Although any tumor may secondarily involve the omentum, the most frequent malignant lesions that metastasize to the omentum include ovarian carcinoma and tumors of the colon and pancreas. Metastases from the stomach, appendix, kidney, uterus and biliary tract may also metastasize to the omentum (22–26). Omental metastasis may be involved in several pathways, including direct extension along the various contiguous ligaments and hematogenous or peritoneal seeding (22). Therefore, this explains our result that aberrant protein expression of MXRA5 was significantly correlated with the lesion site of colon and omental metastases. In addition, omental metastasis was a significant, poor prognostic factor for endometrioid adenocarcinoma, suggesting the need for intra-operative examination of the omentum by close inspection and palpation as well as pathologic examination (27). The significance of the correlation between MXRA5 protein expression and greater omental metastases of CRC requires further investigation.

In this study, our results indicate that MXRA5 protein expression is aberrantly detected in CRC tissues, and has potential value as a biomarker for the early detection of CRC and omental metastasis. However, the reasons for the inconsistency between protein expression and mRNA expression require further study.

## Figures and Tables

**Table I.** Correlation of MXRA5 protein expression with CRC patients’ pathological features.

Clinical features	No. of cases	MXRA5 positive	P-value
Gender	156		0.791
Male	88	60 (68.2%)	
Female	68	45 (66.2%)	
Age (years)	156		0.400
≤60	54	34 (63.0%)	
>60	102	71 (69.6%)	
Lesion sites	156		**0.026**
Colon	75	57 (76.0%)	
Rectum	81	48 (59.3%)	
Gross pathology	156		0.471
Exophytic	43	29 (67.4%)	
Exophytic and ulceration	6	3 (50.0%)	
Ulceration	97	68 (70.1%)	
Infiltrative	10	5 (50.0%)	
Tumor diameter	156		0.470
≤5 cm	89	62 (69.7%)	
>5 cm	67	43 (64.2%)	
Differentiation	156		0.818
Well	14	10 (71.4%)	
Moderate	132	91 (68.9%)	
Poor	10	6 (60.0%)	
TNM staging	156		**0.032**
I and II	55	31 (56.4%)	
III and IV	101	74 (73.3%)	
Invasion	156		0.999
T1	3	2 (66.7%)	
T2	42	28 (66.7%)	
T3	21	14 (66.7%)	
T4	90	61 (67.8%)	
Lymph metastasis	156		0.082
N0	55	31 (56.4%)	
N1	47	33 (70.2%)	
N2	54	41 (75.9%)	
Metastasis	156		**0.043**
Present	70	53 (75.7%)	
Liver metastasis	31	21 (67.7%)	
Omental metastasis	34	28 (82.4%)	
Pelvic cavity metastasis	24	17 (70.8%)	
Lung metastasis	5	1 (20.0%)	
Not present	86	52 (60.5%)	

CRC, colorectal cancer. Bold P-value denotes a statistically significant difference.

**Table II.** Classification of MXRA5 protein immunoreactivity in the CRC tissues, their corresponding normal tissue and the adenoma tissues.

		IRS (%)					P-value	
Group	N	0	1–4	5–8	9–12	All positive	vs. N	vs. Ad
N	156	131 (84%)	25 (16%)	0 (0%)	0 (0%)	25 (16%)		
A	20	7 (35%)	10 (50%)	3 (15%)	0 (0%)	13 (65%)	<0.001	
CRC	156	0 (0%)	51 (33%)	73 (47%)	32 (20%)	156 (100%)	<0.001	<0.001

N, normal mucus; A, adenoma; CRC, coloretal cancer; IRS, immunoreactive score.
